# Consumer Knowledge, Attitude, and Practice Towards Food Package Labels in South Chennai, Tamil Nadu: A Cross-Sectional Study

**DOI:** 10.7759/cureus.91217

**Published:** 2025-08-29

**Authors:** Praveen Murugesan, Anantha Eashwar V.M., Angeline Grace, Aljin V.

**Affiliations:** 1 Community Medicine, Sree Balaji Medical College and Hospital, Chennai, IND

**Keywords:** consumer behavior, food labelling, knowledge-attitude-practice, nutrition policies, urban population

## Abstract

Background

Food packaging labels are vital sources of information that encourage dietary decisions based on knowledge. The way consumers interact with these labels is still not completely understood in urban India, where processed food consumption is growing along with diet-related disease prevalence. This aim of this study is to assess the knowledge, attitude, and practice (KAP) of adult consumers towards food package labels in South Chennai, Tamil Nadu.

Methodology

This cross-sectional study was conducted over 10 months in South Chennai, Tamil Nadu. Three malls were selected using simple random sampling, and 415 adults (≥18 years, residing ≥1 year) were recruited through systematic random sampling (every third eligible adult at the supermarket checkout). A pretested, structured questionnaire in English and Tamil collected sociodemographic data and assessed KAP regarding food labeling. Knowledge responses were scored (correct=1, incorrect/don’t know=0) with ≥50% classified as good. Attitude was recorded on a five-point Likert scale and categorized using the median split. Practice was scored based on frequency and yes/no items, with ≥60% considered good. KAP scores were thus categorized as adequate/inadequate, good/positive, or poor/negative. Data analysis was performed using SPSS v25, with descriptive statistics, chi-square tests, and multivariate logistic regression (odds ratio (OR); 95% confidence interval (CI); p<0.05).

Results

Among the 415 respondents, 66.3% possessed adequate knowledge, 61.7% held positive attitude, and only 51.8% showed good practice in reading labels. Although 85.8% were cognizant of food labels and 70.1% used them during shopping, only 40.2% ever attempted to measure caloric content while 32.1% reported label-reading as a common practice. This suggests a lack of nutritional literacy among the population. In comparison with detailed nutritional information, safety elements like expiry dates were prioritized. Education (adjusted OR (AOR): 0.49; 95% CI: 0.29-0.81; p=0.006) and employment status (AOR: 0.38; 95% CI: 0.23-0.63; p<0.001) were strong predictors of having good knowledge. There were positive relationships in knowledge and attitude (p<0.001) and knowledge and practice (p<0.001), advocating that more knowledge leads to significantly better attitude and behavior.

Conclusion

Consumers in South Chennai demonstrate substantial knowledge and positive attitude towards food package labels; however, a notable gap exists in their practical application, particularly concerning detailed nutritional information, with safety cues often prioritized. Education and employment are crucial determinants of knowledge. These findings underscore the need for targeted, simplified interventions and public health strategies to enhance nutritional literacy and promote informed dietary choices in urban settings.

## Introduction

Food labels serve as an essential public health tool to combat the growing burden of non-communicable diseases (NCDs), particularly in rapidly transitioning nations such as India, where dietary patterns are shifting toward processed and packaged foods [1]. By including details on ingredients, safety, and nutritional value, these labels aim to assist consumers in choosing healthier food. However, according to several studies, consumers in low- and middle-income nations have struggled to understand and implement this information, largely because of low levels of literacy, poor comprehension, and a lack of understanding of nutrition [2]. Vemula et al. studied 1,832 supermarket shoppers in New Delhi and Hyderabad, and found that while nearly 90% of consumers reported reading food labels, the majority restricted their attention to basic details such as manufacturing or expiry dates. Importantly, only about 51% looked at the "Nutrition Facts" panel and 37% reviewed the ingredient list, highlighting that even among urban shoppers, the depth of label use was limited [3].

In 2017, poor dietary habits were linked to around 11 million deaths worldwide, mainly due to excessive sodium intake and insufficient consumption of whole grains and fruits [4]. Moreover, although individuals may claim to use food labels, actual usage remains low and inconsistent, often shaped by low health motivation, poor label design, and the complexity of the data presented [5]. Studies have shown that even simplified approaches, such as menu labeling and front-of-pack (FOP) symbols, have a limited impact, as consumer attention is often diverted or inconsistent [6]. Label comprehension challenges persist even among well-educated populations, who report difficulties due to vague language and low visual clarity [7]. For food labeling to be truly effective, it must be accessible, easy to interpret, and appropriate to the general population’s literacy and comprehension levels [8].

Low well-being and nutrition literacy has been consistently associated with poor understanding of food labels and suboptimal dietary choices [9]. In India, where health education varies widely, food labels remain underutilized despite their presence on nearly all packaged foods [10]. Moreover, socioeconomic factors, such as age, income, and education, affect how labels are read and used, ultimately resulting in discrepancies in health-related behaviors and outcomes [11,12]. Although FOP labels are designed to simplify nutritional decisions, they are frequently overlooked or misinterpreted [13].

While several studies have explored general food label awareness in India, limited research has deeply examined the actual practices and barriers among urban consumers in southern metropolitan settings such as South Chennai. Thus, this study aimed to assess consumer knowledge, attitude, and practice (KAP) of food package labeling in South Chennai, Tamil Nadu.

## Materials and methods

Study design

This study employed an analytical cross-sectional design to investigate consumer KAP regarding food package labeling in South Chennai, Tamil Nadu. The research targeted adults aged ≥18 years who had resided in South Chennai for at least one year, focusing on supermarket shoppers to capture authentic insights into label engagement.

Study setting

The study was conducted over a 10-month period (June 2024-March 2025) in supermarkets located inside three major malls in South Chennai. The malls were selected using simple random sampling.

Inclusion and exclusion criteria

Adults aged ≥18 years, residing in South Chennai for ≥1 year, who purchased packaged foods from the selected supermarkets, were included in the study.

Individuals who primarily purchased packaged foods online or from local stores, or those unwilling to participate were excluded. This exclusion limited generalizability to supermarket shoppers.

Sample-size calculation

The sample size was calculated based on a previous study by Robert and Chandran, which reported that 46% of South Indian consumers read food labels [1]. The required sample size was 382 as per the formula:

$n = \frac{Z^2 \cdot p \cdot q}{l^2}$

with Z=1.96 (95% confidence interval (CI)), p=0.46, q=0.54, and margin of error (l)=5%. Accounting for a 5% non-response, the target was 403, and ultimately, 415 participants completed the survey.

Sampling technique

Within each selected mall, participants were recruited using systematic random sampling. Every third eligible adult exiting the supermarket checkout counter was invited to participate. If an individual was ineligible or declined, the next eligible person was approached. This approach minimized selection bias and ensured representation of a diverse consumer population.

Data collection tool

A pretested, structured questionnaire was developed based on existing literature, with modifications for local relevance. It was first translated into Tamil and then back-translated into English by bilingual experts to ensure clarity and cultural appropriateness. The questionnaire was piloted with 20 participants (not included in final analysis), and necessary adjustments were made based on their feedback. Data were collected via face-to-face interviews conducted by trained investigators in either Tamil or English, based on participant preference.

The questionnaire included sociodemographic information (age, gender, education, occupation, and income); knowledge of food package labeling (each correct “Yes” response was scored as 1; incorrect or “Don’t know” responses were scored as 0. Total scores were converted to percentages, with ≥50% classified as good knowledge and <50% as poor knowledge); attitude toward food labels (responses were recorded on a five-point Likert scale (1=strongly disagree to 5=strongly agree), with favorable responses receiving higher scores. Overall scores were categorized as positive or negative using the median split.); practice related to label use (frequency-based responses were scored (regularly/always=1, sometimes/rarely/never=0), and Yes/No items were scored (Yes=1, No=0). Participants achieving ≥60% of the maximum practice score were categorized as having good practice, and <60% as poor practice.). The structured questionnaire used in this study is provided in the appendix.

Data collection procedure

Data collection was carried out across 14 visits distributed over weekdays and weekends to ensure diversity. Participants were fully informed about the study objectives, procedures, and voluntary nature before obtaining written consent.

Data analysis

Collected data were entered into Microsoft Excel and analyzed using SPSS version 25 (IBM Corp., USA). Descriptive statistics summarized sociodemographic profiles and KAP. Chi-square tests and multivariate logistic regression (odds ratios (ORs) with 95% CIs) were used to examine associations. Statistical significance was set at p<0.05.

Ethical considerations

Before data collection, ethical approval was obtained from the institutional ethical committee of a tertiary medical college. No personal identifiers were recorded, and participants were assigned unique IDs to maintain confidentiality. Written informed consent was obtained, and participants had the right to decline or withdraw at any time without consequences. Participation was completely voluntary, and participants’ autonomy, privacy, and confidentiality were respected throughout the study.

## Results

Sociodemographic characteristics

The sociodemographic characteristics of the study participants are provided in Table 1. The age distribution of the population significantly differed, with the highest percentage (59.0%) of the respondents being between the ages of 21 and 40, and the next highest percentage (28.4%) being between the ages of 41 and 60. With 50.4% of the population being male and 49.6% being female, the gender distribution was almost equal. With respect to education, 80.0% of participants were employed, and the majority of them (81.0%) were literate. Most of participants (60.7%) were married. The majority of households (55.7%) had four to six individuals. The urban economic landscape of the sample was seen in the fact that 33.0% of participants stated they had a monthly income between Rs 25,000 and Rs 50,000

**Table 1:** Sociodemographic characteristics of the study participants

Variable	Category	Frequency (n=415)	Percent %
Age	18-20	46	11.1
	21-40	245	59
	41-60	118	28.4
	>60	6	1.4
Gender	Female	206	49.6
	Male	209	50.4
Education	Illiterate	79	19.0
	Literate	336	81.0
Occupation	Unemployed	83	20.0
	Employed	332	80.0
Household Members	>6	51	12.3
	1-3	133	32
	4-6	231	55.7
Marital Status	Married	252	60.7
	Other	3	0.7
	Separated	20	4.8
	Unmarried	127	30.6
	Widowed	13	3.1
Monthly Income	Less than Rs 25,000	98	23.6
	Rs 25,000-50,000	137	33
	Rs 51,000-75,000	113	27.2
	More than Rs 75,000	67	16.1

**Figure 1:**
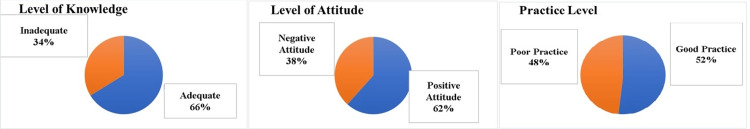
Level of KAP KAP: Knowledge, attitude, and practice

Figure 1 illustrates the overall levels of KAP regarding food package labeling among the study participants. A substantial proportion of participants (66.3%, n=275) demonstrated adequate knowledge, particularly regarding the link between processed foods and health hazards. Regarding attitude, 61.7% (n=256) of respondents reported a positive attitude, indicating that they found labels helpful in making healthier dietary decisions. However, a notable finding was that only 18.8% of respondents expressed full trust in the nutritional information provided on the labels. In terms of practice, 51.8% (n=215) of the participants reported good practice, with 70.1% indicating that they chose products based on labels and 67.5% avoiding unlabeled items. Despite these positive indicators, only 40.2% of participants utilized labels to calculate their caloric consumption, and only 32.1% reported frequently reading them. Expiry dates, vitamins, and trans fats were among the most frequently examined items on food labels, suggesting a prioritization of safety and specific nutrients over comprehensive nutritional understanding by consumers.

Table 2 presents the associations between participants’ demographic characteristics and their level of knowledge regarding food package labels. There was a statistically significant correlation (p=0.013) between knowledge and educational status, suggesting that those with formal education were more likely to possess an adequate understanding. As seen by the strong correlation between occupation and knowledge levels (p<0.001), the employed tended to have higher knowledge levels. However, no statistically significant correlation was found between knowledge levels and other demographic characteristics, including age (p=0.344), gender (p=0.351), household size (p=0.386), marital status (p=0.238), and monthly income (p=0.468).

**Table 2:** Association between demographic variables and knowledge levels

Variables	Adequate Knowledge		Inadequate Knowledge		p-value
	n	%	n	%	
Age					
≤40 years	197	71.6	94	67.1	0.344
>40 years	78	28.4	46	32.9	
Gender					
Male	134	48.73	75	53.57	0.351
Female	141	51.27	65	46.43	
Education					
Illiterate	43	15.6	36	25.7	0.013
Literate	232	84.4	104	74.3	
Occupation					
Employed	234	85.1	98	70	<0.001
Unemployed	41	14.9	42	30	
Household Members					
1-3	85	30.91	48	34.29	0.386
4-6	152	55.27	79	56.43	
>6	38	13.82	13	9.29	
Marital Status					
Married	169	61.45	83	59.29	0.238
Separated	14	5.09	6	4.29	
Widowed	9	3.27	4	2.86	
Unmarried	83	30.18	44	31.43	
Other	0	0	3	2.14	
Monthly Income					
Less than Rs 25,000	71	25.82	27	19.29	0.468
Rs 25,000-50,000	87	31.64	50	35.71	
Rs 51,000-75,000	75	27.27	38	27.14	
More than Rs 75,000	42	15.27	25	17.86	

Table 3 shows the independent effects of occupation and education on knowledge levels were further supported by multivariate logistic regression analysis, which is shown in Table 3. Literacy was a major predictor of food label knowledge, as evidenced by the significantly reduced odds of acceptable knowledge among illiterate participants (adjusted OR (AOR)=0.49; 95% CI: 0.29-0.81; p=0.006) compared with literate participants. Comparing unemployed and employed participants, the former showed significantly lower odds of having appropriate knowledge (AOR=0.38; 95% CI: 0.23-0.63; p<0.001), highlighting the crucial impact of employment status on information availability and general awareness.

**Table 3:** Multivariate logistic regression analysis for demographic factors and knowledge level OR: Odds ratio; CI: Confidence interval

Variables	Knowledge Level OR (95% CI)	p-value
Education: Literate, Illiterate	Ref 0.49 (0.29-0.81)	0.006
Occupation: Employed, Unemployed	Ref 0.38 (0.23-0.63)	<0.001

Table 4 indicates that attitude toward and knowledge of food packaging labels are strongly and statistically significantly correlated (p<0.001). Among participants with limited knowledge, 55.4% had a positive attitude, whereas 73.1% of those with strong knowledge demonstrated a positive attitude. Percentages are calculated column-wise, showing that higher knowledge is associated with a more positive attitude toward food package labels.

**Table 4:** Association between attitude and knowledge level

Knowledge	Attitude		p-value
	Positive (n (%))	Negative (n (%))	
Good	187 (73.05)	88 (55.35)	<0.001
Bad	69 (26.95)	71 (44.65)	

Table 5 shows a significant association between knowledge and practice (p<0.001). Percentages in Table 5 are calculated column-wise. Within each knowledge category, the proportion of participants reporting good practice was determined, 67.4% of participants with good knowledge reported good practice, compared to 65.7% of those with poor knowledge, indicating how practice levels vary across knowledge groups

**Table 5:** Association between knowledge and practice

Knowledge	Practice		p-value
	Good (n (%))	Bad (n (%))	
Good	180 (67.4)	95 (65.7)	<0.001
Bad	35 (32.6)	105 (34.3)	

This study revealed a complex interplay of knowledge, attitude, and practice concerning food package labels among consumers in South Chennai. Although a commendable proportion of the population demonstrated adequate knowledge and positive attitudes, particularly regarding the health implications of processed foods and the general helpfulness of labels, a noticeable gap exists in the practical application of this information. Consumers tend to prioritize immediate safety cues, such as expiry dates, over detailed nutritional content, and the actual calculation of calorie intake from labels remains low. Education and occupation emerged as crucial sociodemographic determinants of knowledge, highlighting disparities in awareness based on these factors.

Furthermore, while adequate knowledge significantly fosters positive attitude, the translation of both knowledge and positive attitudes into consistent and comprehensive label-reading practices is not as robust. This suggests that simply providing information may not be sufficient; other factors, such as label complexity, time constraints, or lack of perceived relevance for specific nutrients, might impede the full utilization of food labels. 

## Discussion

The current study explored the KAP of adults who shopped in South Chennai supermarkets with focus on food product labeling. The results showed that 51.8% of participants used proper label-reading methods, 66.3% of participants had adequate knowledge, and 61.7% had positive attitudes. Although this indicates an acceptable level of awareness, there is still a clear disconnection between information and practical behavioral application in eating choices.

Similar trends have been noted internationally, according to research done on students in China and Saudi Arabia, while many people noticed food labels, only a few of them understood or implemented them well when making decisions [14,15]. Studies conducted in India, such as those conducted in Puducherry and other metropolitan areas, have also shown that although awareness is there, label-based purchasing decisions are rarely taken [16,17].

It's interesting to note that, in contrast to our study, Robert and Chandran observed that only 5% of their participants had enough knowledge [1]. The results we obtained indicated a more direct relationship between educational status and knowledge levels, even though they reported a stronger association between education and label-reading frequency. This can be a result of higher knowledge, understanding of urban health issues, and exposure to Chennai's marketing initiatives.

The results of our study corresponded with the findings of Vemula et al., who reported that about 25% of respondents experienced difficulties in understanding labels due to factors such as small font size, technical terminology, or unclear layout [3]. While our participants also mentioned legibility concerns in their feedback, these were not quantified in percentage terms in our dataset. Participants in our study also reported challenges with the legibility of food labels, particularly small font size and technical terms, though these were not quantified separately.

In Western countries, systematic reviews consistently show that interpretative shapes, including color codes or FOP symbols, are more effective than normal numeric tables at influencing customer decisions [18-20]. Interpretation and use of label information are further influenced by elements such as literacy, personal interests, and the shopping environment.

Studies such as Baig et al., which noted that though healthcare students in Mangalore had moderate knowledge of food labeling and recognized the importance of expiry dates and nutrition, few altered their food preferences accordingly, and Veena Suresh et al., where only 20% of students achieved a good KAP score and commonly prioritized expiry date, price, and vegetarian status over nutritional details, support our findings [21,22]. In our study, while 90% of participants avoided products without labels, many admitted to focusing more on price and expiry date than on nutritional content. Similar findings were made by Bhattacharya et al., who noted that although knowledge is high, interpretation is still low, which results in a preference for simpler formats such as traffic-light labels or icons [23]. Furthermore, it was shown by Graham and Laska that increased usage of food labels for making food purchase and dietary decisions is positively correlated with healthier eating practices, particularly among people who are educated and health-conscious [24]. In the present study, 52% of respondents indicated they regularly use food labels when choosing or buying food products, exceeding the 45% noted in the Mysuru study conducted by Krishnaraj et al. [25]. This positive trend could indicate an increased awareness of health and greater familiarity with packaged food in urban areas such as South Chennai.

Limitations

This cross-sectional study cannot determine causality or track changes in KAP over time. The sample includes only adults visiting selected supermarkets in South Chennai, excluding online or neighborhood store shoppers, which may limit generalizability and introduce selection bias. Findings may not apply to rural populations or other regions. Reliance on self-reported data could cause recall or social desirability bias. The study also does not assess the impact of specific label designs or regulatory changes. Despite these limitations, it provides valuable region-specific insights into consumer engagement with food labels.

Recommendations

The study's conclusions suggest several actions to help consumers better understand and use food package labels. First, using FOP images with traffic light symbols or star ratings can reduce confusion and improve the clarity of food labels. These designs help consumers quickly grasp important nutritional information without having to read long panels. Second, public health campaigns should do more than just raise awareness, they should also teach people how to read labels correctly, including how to check a food item’s calorie, fat, sugar, and salt content. Third, educating students on how to read food labels in schools and universities can help them develop healthy eating habits early. Additionally, using digital tools and mobile phone apps can make it easier for people to understand label information, especially while shopping. Together, these efforts can promote community acceptance of healthy eating and improve understanding.

## Conclusions

This study successfully evaluated South Chennai, Tamil Nadu, consumers' KAP regarding food package labeling. The results show that although a significant percentage of the population has knowledge of and has positive thoughts about food labels, there is a significant absence of practical use of these labels, especially when it concerns detailed nutritional data. Knowledge levels have been found to be significantly influenced by education and occupation. In an effort to address the observed knowledge-practice gap and encourage consumers to make more aware and healthier food choices in this urban environment, these findings highlight the urgent need for focused, straightforward interventions and educational initiatives.

## Figures and Tables

**Figure 2:**
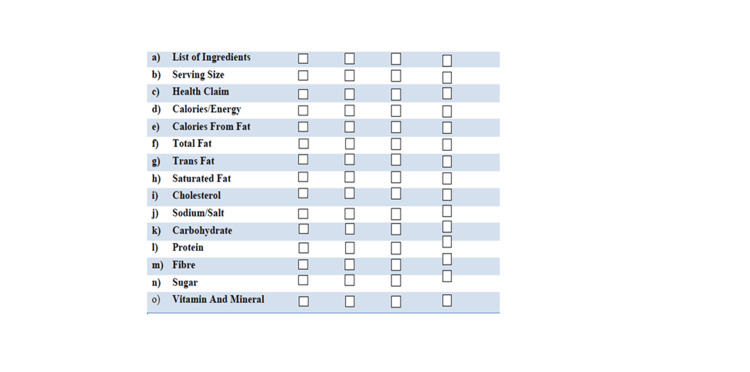
Items included in the food package label

## Appendices

Questionnaire

Part I - Demographic Details

1. Age (years):

a) 18-20 b) 21-40 c) 41-60 d) >60

2. Gender

a) Male b) Female

3.  Education Status (completed):

a) Primary and Middle School b) Higher Secondary c) Graduate d) Post Graduate e) Professional f) Illiterate g) Other

4. Occupation:

a) Professional (ex: doctors, bank managers, engineers) b) Semi professional (ex: teachers, lecturers, junior administrators) c) Skilled worker (ex: clerk, typist, accountant) d) Semi-skilled worker (ex: factory laborer, laboratory or library attendant) e) Unskilled worker (ex: watchman, peon, domestic servant) f) Unemployed

5. Total number of house hold members:

a) 1-3 b) 4-6 c) >6

6. Marital status:

a) Married b) Unmarried c) Widowed d) Separated e) Other:

7. Total monthly income of the family:

a) Less than Rs 25000 b) Rs 25,000-50,0000 c) Rs 51,000-75,000 d) More than Rs 75,000

Part II - Knowledge Regarding Food Package Labeling

1. Do you know food items come with food package labeling?

a) Yes b) No

2. Do you know what purpose these food package labeling serve to consumers?

a) Yes b) No

3. Where are these food package labels usually present on the food items?

a) Front side b) Back side c) Sides d) Don't Know

4. Do use of these packed processed foods lead to any disease?

a) Yes b) No c) Don't know

Part III - Attitude and Perceptions Towards Food Package Labeling

1. I found nutrition menu label helpful.

a) Strongly disagree b) Disagree c) Neutral d) Agree e) Strongly agree

2. It is interesting for me to read the nutrition menu label always.

a) Strongly disagree b) Disagree c) Neutral d) Agree e) Strongly agree

3. I would use calorie information to order low-calorie foods and drinks.

a) Strongly disagree b) Disagree c) Neutral d) Agree e) Strongly agree

4. It is beneficial for me to read the nutrition menu label.

a) Strongly disagree b) Disagree c) Neutral d) Agree e) Strongly agree

5. I trust the nutritional information provided on the nutrition menu label.

(1 - No, 5 - Full trust)

1        2        3        4        5

6. The nutritional information provided affects my decision to purchase.

(1 - doesn't affect, 5 - fully affect)

1        2        3        4        5

7. Which do you think is very important in a food label?

a) Price b) Expiry date c) Ingredients d) Nutrition information e) Brand name

8. I believe nutritional information should not be misleading.

a) Strongly disagree b) Disagree c) Neutral d) Agree e) Strongly agree

9. Nutritional information (carbohydrate, protein, fat) indicated by percentage is sufficient for me to know how many ingredients the food contains.

a) Yes b) No c) Need additional information

Part IV - Practices on Food Package Labels

1. How often do you buy packed/processed food items?

a) Daily b) Sometimes in a week c) Rare d) Never

2. Why do you buy packed foods? (multiple responses)

a) Cheap b) Easily available c) Tasty d) Ready to eat e) Accessible f) Not applicable

3. How often do you read food package labels?

a) Regularly b) Sometimes c) Never

4. How often do you notice/read the following items in the food package label (see example in Figure 2)?

5. Will you choose the product to buy based on the information provided in the food package label?

a) Yes b) No

6. Will you avoid buying a food product if there is no food package label?

a) Yes b) No

7. Do you calculate your calorie intake based on the information given in the food label?

a) Yes b) No

Thank you for participating in the survey.
